# A snare technique for valve-in-valve transcatheter aortic valve replacement in a stented coarctation of the aorta: a case report

**DOI:** 10.3389/fcvm.2026.1685948

**Published:** 2026-04-29

**Authors:** Juyong Lee, Mahmoud Barbarawi, Alessandra Bassani, Kyutae Park, Supriya Tigadi, Agnes S. Kim, Chittoor B. Sai Sudhakar

**Affiliations:** 1 Department of Cardiovascular Medicine, Section of Interventional Cardiology, University of Connecticut School of Medicine, Farmington, CT, United States; 2 Department of Cardiovascular Medicine, Parrish Medical Center, Titusville, FL, United States; 3 University of Connecticut School of Medicine, Farmington, CT, United States; 4 Chuncheon Sacred Heart Hospital, Hallym University College of Medicine, Gangwon-do, Chuncheon, Republic of Korea; 5 Department of Cardiothoracic Surgery, HCA Florida Largo Hospital, Largo, FL, United States

**Keywords:** coarctation of the aorta, prosthetic aortic valve stenosis, transcatheter aortic valve replacement (TAVR), Turner syndrome, valve-in-valve TAVR

## Abstract

**Background:**

Self-expanding valves are effective for transcatheter aortic valve replacement (TAVR); however, device delivery can be challenging in patients with a distorted aortic anatomy. Herein, we report the successful use of a snare technique to facilitate valve delivery in a patient with a previously stented aortic coarctation and poststenotic dilatation.

**Case summary:**

A 66-year-old woman with Turner syndrome, prior aortic coarctation stenting, and surgical bioprosthetic aortic valve replacement presented with symptomatic severe prosthetic aortic valve stenosis. Advancement of a 23-mm Medtronic Evolut FX valve was hindered by the small caliber of the patent aortic stent and marked aortic tortuosity. A gooseneck snare was used to apply inferior traction while the delivery system was advanced from the contralateral femoral access, redirecting the catheter toward the center of the stented segment and enabling successful valve deployment.

**Conclusion:**

Snare-assisted redirection represents a valuable adjunctive strategy to facilitate self-expanding TAVR valve delivery in patients with complex or previously stented aortic anatomy. Durable valve function without complications was observed in the patient at a 2-year follow-up.

## Introduction

Transcatheter aortic valve replacement (TAVR) is an established therapy for patients with symptomatic severe aortic stenosis (AS) who are at increased surgical risk and has demonstrated non-inferiority to surgical aortic valve replacement in selected patients at low surgical risk (1, 2). Self-expanding bioprosthetic valves, such as the Medtronic Evolut FX (Minneapolis, MN, USA), have been shown to be safe and effective for the treatment of AS (3). However, delivery of self-expanding systems can be challenging in patients with complex aortic anatomy, including marked tortuosity, prior aortic interventions, or a horizontal aortic root. These anatomical features may increase the risk of procedural complications such as valve malposition, aortic injury, the need for postdilation, and paravalvular leak (4, 5).

In this study, we describe the case of a valve-in-valve TAVR in a patient with prior aortic coarctation stenting and poststenotic dilatation and in whom a snare technique enabled successful delivery of a self-expanding valve.

## Case presentation

**Figure 1:**
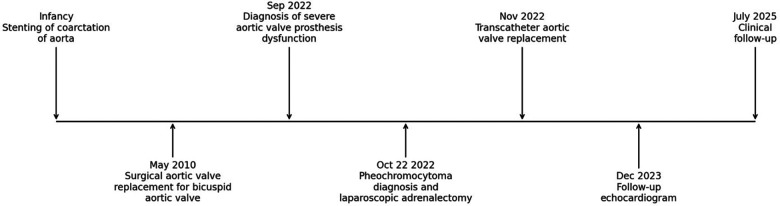
A timeline summarizing key clinical events, interventions, and follow-up.

A timeline summarizing key clinical events, interventions, and follow-up is provided in Figure 1.

A 66-year-old woman with Turner syndrome, coarctation of the aorta treated with endovascular stenting early in life, coronary artery disease status postpercutaneous coronary intervention, chronic atrial fibrillation, recurrent gastrointestinal bleeding, and bicuspid aortic valve stenosis status postsurgical aortic valve replacement with a 21 mm ThermaFix pericardial valve (Edwards Lifesciences, Irvine, CA, USA) in 2010 presented with progressive dyspnea that limited her daily activities. She was referred for evaluation and management of symptomatic severe prosthetic aortic valve stenosis.

During the preoperative evaluation for redo surgical aortic valve replacement, an incidental right adrenal mass was identified on imaging. A biochemical testing revealed elevated plasma normetanephrine and metanephrine levels, consistent with pheochromocytoma. A laparoscopic adrenalectomy was performed and completed without any complication.

Following adrenalectomy, a multidisciplinary heart team discussion determined that transcatheter valve-in-valve aortic valve replacement was preferable over redo surgical intervention given the patient's high-risk features, and a shared decision was made to proceed with TAVR.

**Figure 2:**
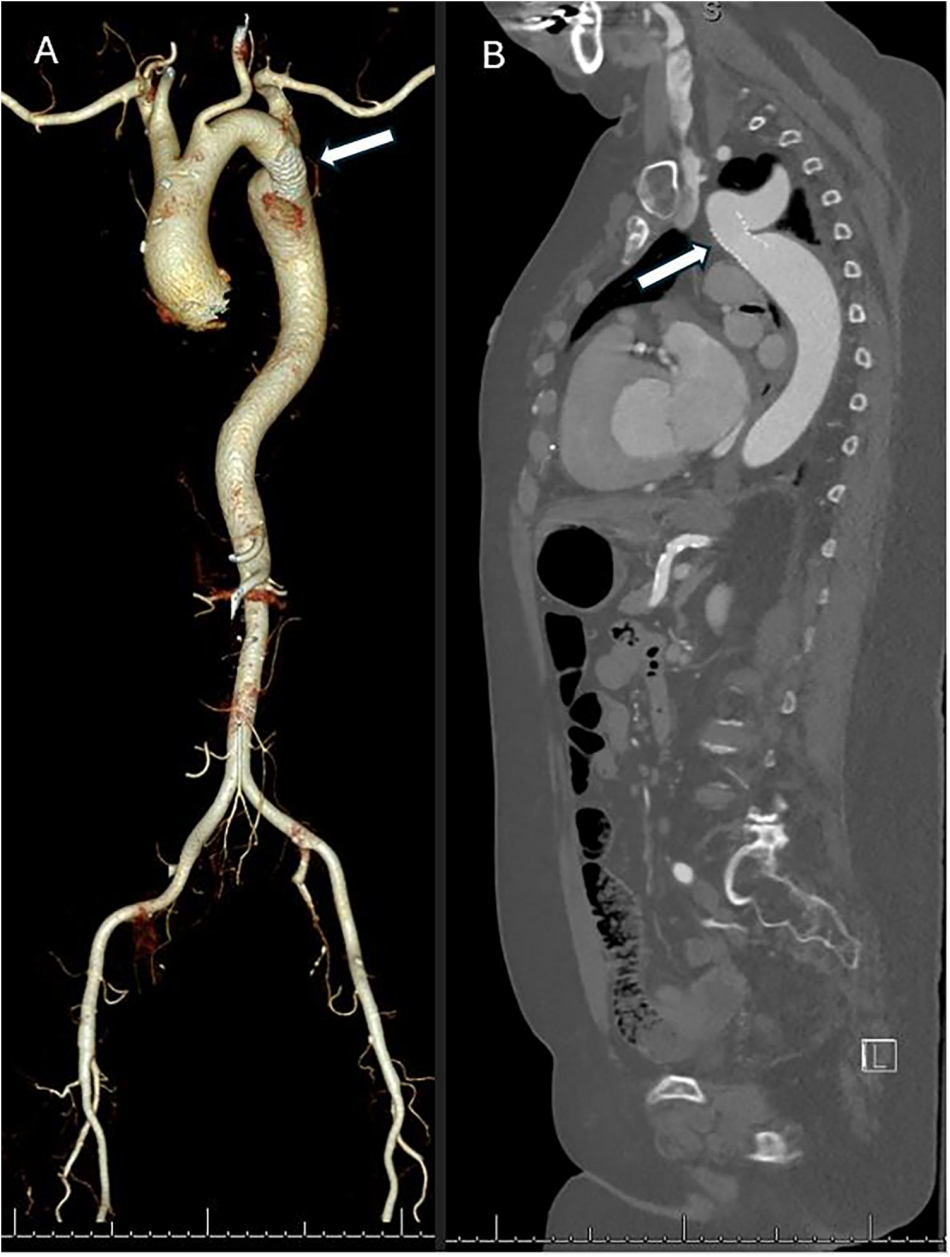
A preprocedural computed tomography demonstrating complex aortic anatomy. **(A)** A three-dimensional volume-rendered CT reconstruction shows marked aortic tortuosity with poststenotic dilatation of the descending thoracic aorta. The previously placed aortic coarctation stent is indicated by the white arrow. **(B)** A sagittal CT angiography demonstrates the stented coarctation segment (white arrow) with a small luminal diameter and pronounced poststenotic dilatation, explaining the difficulty in advancing the transcatheter valve delivery system.

A transthoracic echocardiography demonstrated severe prosthetic valve stenosis, with a peak transvalvular velocity of 4.5 m/s, a mean gradient of 54 mmHg, an acceleration time of 146 ms, and a velocity-time integral ratio of 0.22, corresponding to an effective orifice area of 0.7 cm². Left ventricular ejection fraction was mildly reduced at 40%. A preprocedural computed tomography angiography demonstrated a patent aortic stent just distal to the left subclavian artery with a minimum luminal diameter of 13 mm, marked poststenotic dilatation of the descending thoracic aorta (3.3 cm), and significant aortic tortuosity (Figures 2A,B), explaining the anticipated difficulty in advancing the transcatheter valve delivery system.

A bilateral common femoral arterial access was obtained. A 23 mm Medtronic Evolut FX valve was introduced via the right common femoral artery and advanced over a Safari wire. Multiple attempts to cross the stented segment were unsuccessful due to abutment between the delivery system and the stent struts in the setting of severe tortuosity. The wire position along the greater curvature of the aorta further biased the delivery trajectory toward the aortic wall and stent.

**Figure 3:**
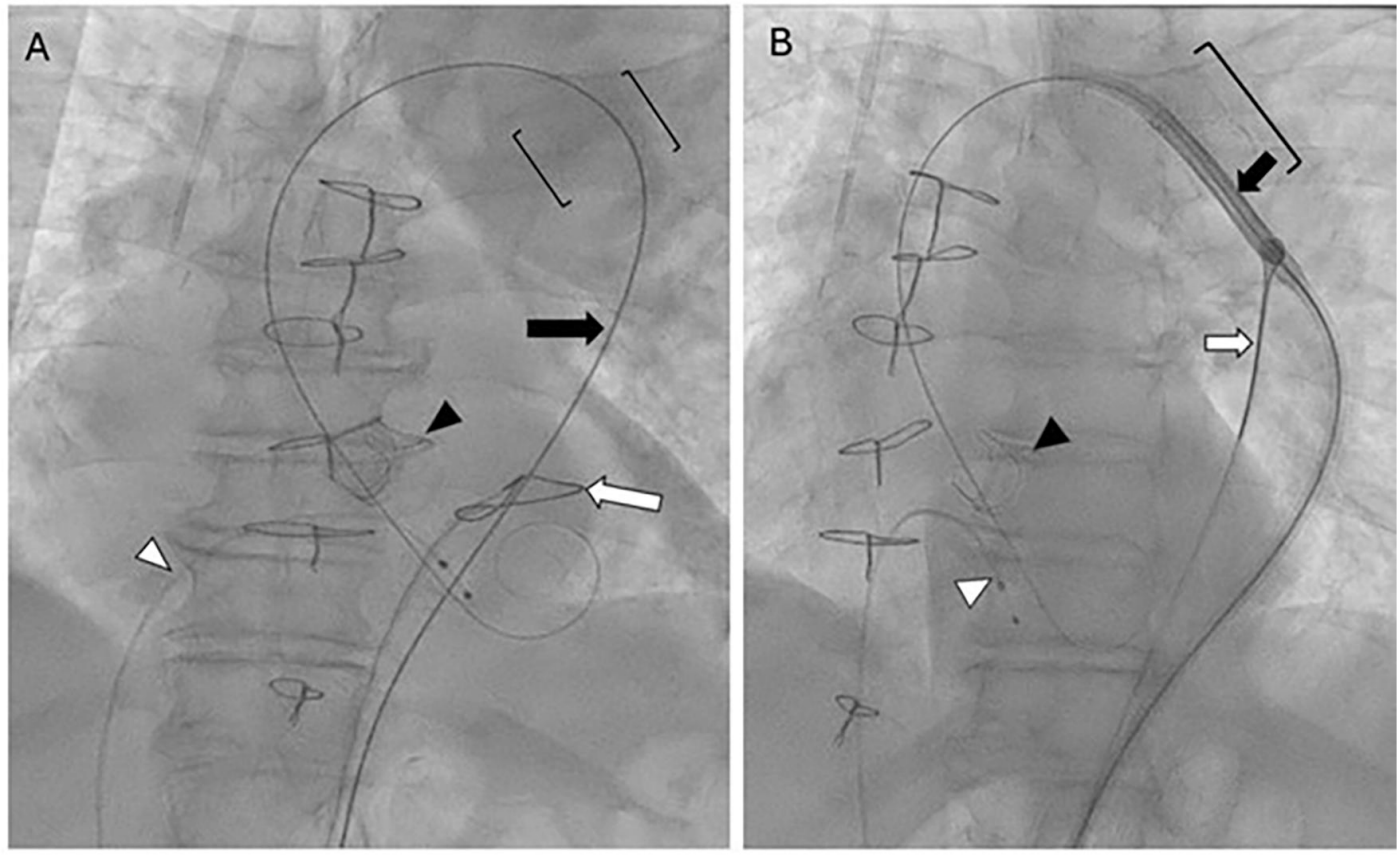
A snare-assisted delivery of the TAVR system across a stented coarctation. **(A)** Fluoroscopy demonstrates a gooseneck snare (white arrow) advanced over a JR4 catheter, with the Safari wire (black arrow) passed through the center of the snare loop across the stented aortic segment (brackets). The prior surgical bioprosthetic aortic valve is indicated by the black arrowhead, and the pacemaker lead is denoted by the white arrowhead. **(B)** Fluoroscopy shows the gooseneck snare (white arrow) tightened around the valve delivery catheter (black arrow), enabling passage across the stented aortic segment (bracket). The prior surgical bioprosthetic valve (black arrowhead) and pacemaker lead (white arrowhead) are also shown.

To facilitate delivery, a snare technique was employed (Supplementary Video). The valve delivery system was withdrawn, and a 14-Fr sheath was placed in the right common femoral artery. Through the left common femoral artery, a large gooseneck snare was advanced via a 6-Fr Judkins Right-4 catheter into the abdominal aorta (Figure 3A). The Safari wire was passed through the snare loop and advanced into the left ventricle. The Evolut FX delivery system was then reintroduced from the right access and advanced through the snare. The snare was intentionally applied to the proximal shaft of the delivery catheter rather than the nose cone. Shaft-based snaring allowed controlled, coaxial redirection of the delivery system toward the center of the stented segment while minimizing excessive angulation and focal stress at the distal tip. Capturing the nose cone in this setting could have exaggerated catheter angulation within the rigid stented segment, increased the risk of buckling, or interfered with normal device mechanics, potentially leading to delivery system malfunction or impaired recapture capability in a severely tortuous anatomy.

**Figure 4:**
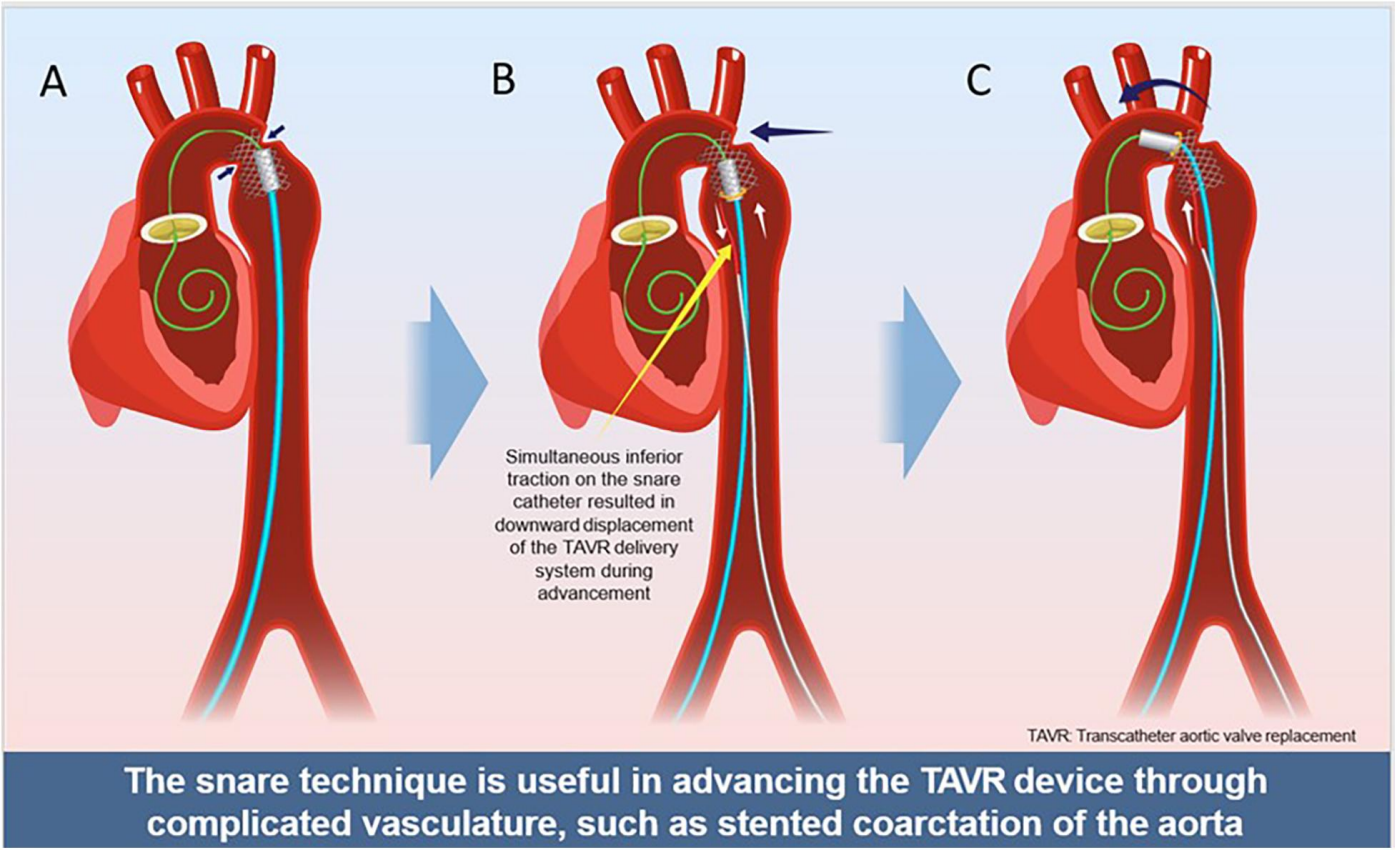
**(A)** Initial advancement of the TAVR delivery catheter was unsuccessful due to a small, previously placed aortic stent and marked aortic tortuosity. **(B)** A Safari wire was advanced through an open gooseneck snare and positioned across the degenerated surgical bioprosthetic valve. The snare was tightened around the delivery catheter, and coordinated forward advancement with inferior traction redirected the system toward the center of the stented segment. **(C)** The delivery catheter successfully crossed the stented segment into the aortic arch.

Once positioned in the descending thoracic aorta, the snare was tightened around the proximal shaft of the delivery catheter (Figure 3B). Coordinated forward advancement from the right groin and inferior traction on the snare from the left redirected the system toward the center of the stented segment, enabling successful passage across the stent into the aortic arch. The snare was subsequently released and withdrawn. The Evolut FX valve was advanced to the level of degenerated surgical bioprosthesis and deployed using the standard technique. A final thoracic aortography demonstrated no aortic injury and no paravalvular leak. A predischarge transthoracic echocardiography confirmed normal transcatheter valve function (Figure 4).

At 1-year follow-up, an echocardiography demonstrated a well-functioning valve with improvement in the left ventricular ejection fraction to 50%. At 2 years, the patient remained clinically stable without recurrence of cardiac symptoms.

## Discussion

This case highlights a technically challenging valve-in-valve TAVR procedure in a patient with prior aortic coarctation stenting, marked tortuosity, and poststenotic dilatation. To our knowledge, there are very limited published reports describing snare-assisted techniques to facilitate delivery of self-expanding TAVR systems through a previously stented and severely tortuous descending thoracic aorta.

The Medtronic Evolut FX valve offers several advantages, including self-expanding deployment, a reduced risk of annular rupture, and a supra-annular leaflet design that may optimize hemodynamics in small surgical bioprostheses. In the case of our patient, a self-expanding valve was selected over a balloon-expandable system due to concerns regarding annular compliance, the small size of the prior surgical valve, and the heightened risk of annular injury due to Turner syndrome and complex aortic anatomy. New-generation self-expanding platforms such as the Navitor valve incorporate design features that may further enhance flexibility and deliverability in anatomically challenging cases and may represent reasonable alternatives depending on device availability and operator experience.

Despite these advantages, self-expanding valve systems can be challenging to advance in patients with a distorted or horizontal aortic anatomy, increasing the risk of procedural failure or vascular injury. Alternative adjunctive techniques such as buddy wires, balloon-assisted tracking, and snare-assisted redirection have been described; however, in the case of the patient in this study, the presence of a rigid prior aortic coarctation stent combined with marked poststenotic dilatation limited effective directional control of the delivery trajectory. Delivery system failure in patients with complex anatomy has been reported, including instances of patients requiring snare bailout techniques (6). These observations underscore the importance of meticulous procedural planning and familiarity with adjunctive maneuvers when performing TAVR in patients with complex aortic anatomy (7, 8).

Alternative access routes such as transcarotid or trans-subclavian approaches represent well-established and often effective strategies for transcatheter aortic valve replacement in patients with unfavorable transfemoral anatomy. In the case of our patient, transfemoral access was initially selected based on preprocedural imaging that demonstrated a patent aortic coarctation stent with acceptable luminal dimensions; however, the degree of delivery system interaction with the rigid stented segment and aortic tortuosity was not fully anticipated. In retrospect, alternative access may have provided a more direct and stable delivery trajectory. Nonetheless, femoral snare-assisted inferior traction allowed successful redirection of the delivery system without the need to change access during the procedure. Importantly, in future cases of patients with similar anatomy—particularly those involving rigid aortic stents combined with marked tortuosity—primary consideration of transcarotid or trans-subclavian access would be strongly favored to optimize coaxial alignment and facilitate safe valve delivery.

In this patient case, shaft-based snaring enabled controlled, reversible redirection of the delivery catheter away from the aortic wall and toward the center of the aortic lumen. By intentionally avoiding nose-cone capture, this approach minimized focal stress at the distal tip of the delivery system and reduced the risk of excessive angulation, device buckling, stent interaction, or delivery system malfunction during advancement. Potential risks of snare use include vascular injury, wire entanglement, and loss of device control; however, these risks can be mitigated with continuous fluoroscopic guidance, gentle traction, and close coordination between operators. The snare was applied transiently and released immediately after successful device passage without any complication. This surgical experience highlights shaft-based snare-assisted redirection as both a facilitative and bailout strategy in anatomically challenging TAVR procedures.

## Conclusion

The snare technique can serve as an effective facilitative and bailout strategy for delivering self-expanding TAVR systems through complex, previously stented, and tortuous aortic anatomy. This patient case broadens the applicability of snare-assisted redirection beyond horizontal aortas and underscores its value in overcoming challenging aortic geometries encountered during valve-in-valve TAVR.

## Data Availability

The original contributions presented in the study are included in the article/Supplementary Material, and further inquiries can be directed to the corresponding author.

## Supplementary material

Supplementary Video Fluoroscopy video illustrates the simultaneous coordinated movement of the valve delivery catheter and the gooseneck snare.
